# Hats and Baldness

**Published:** 1883-04

**Authors:** Ben. H. Pratt


### THE BISTOURY.

Vol. XIX. ELMIRA, N. Y., APRIL, 1883. No. 5. 1

### Contributions.

### HATS AND BALDNESS.

BEN. H. PRATT.

There is much said about tight boots, those corniferous, bunioniferous deformers of feet, inventions of the devil and inflicted upon us by his agent, human vanity. The consequence of tight boots is that there isn't a natural foot in what is called respectable society. But nobody's to blame for tight boots except ourselves. There's no law compelling one to wear a certain size of boot, and we all know precisely what the result is of wearing one too small for the foot. I can't find fault with a man or woman who seems to enjoy torturing himself or herself. It is their personal right—none of my business. They know the effects. They are merely fools. I may pity them—do, in fact—and when they go limping about inquiring for chiropodists and salves and plasters and ointments and charms, it's natural for the most sympathetic in all ordinary ills to smile at this sort of ill, and yearn for the dawn of that blessed day when either big shoes shall be *a la mode*, or people will learn the glorious enjoyment of uncramped feet and cease to be governed by their mania for delicate pedal formations.

But there's another article of apparel which men wear tight, not because it looks better tight, or feels better tight, or has any particular advantage whatever in being tight. It is the hat. The majority of men, however unconscious they may have become of the fact, wear their hats too tight. I don't know why. It is probably from habit alone. When men talk together with their hats on, watch them. They will change the location of the hat about as often as they transfer the weight of the body from one leg to the other. I expect man was never intended to be hatted or capped or even turbaned. It is more than probable that his hair was given him for a protection against heat and cold. If not, what in the world is it for? Ornament? It's no use speculating as to the design of creation in providing hair, and it is also useless, even absurd, in the present advanced prejudice of the civilized world, to expect that hats will ever be done away with by men. Man seems to be destined to cover every coverable part of his body, and our noses and ears would doubtless have been provided with their respective coverings long ago, if it were not so provokingly inconvenient. It seems to me to be a sort of oversight in the compilation of human senses that some office or function was not bestowed upon the hair that would have compelled us to keep it uncovered. If, for instance, the hair had been endowed with the function of detecting the presence of material objects when near them, a function which the hair of the head, with its poly-pointing directions, seems admirably adapted for, we wouldn't be covering up our heads and making ourselves uncomfortable, and bringing about diseases of the scalp, and loss of hair, and no doubt mental depreciation through the unnatural pressure. But nature has given us a chance to cover up our heads and we've taken the chance, and right around the heads of nine men out of ten there is a line of demarkation, separating a well studded growth of hair from a more or less thinned out portion, the degree of thinness varying from a just observable difference on opposite sides of the line, to a clearly defined division of heavy hair and absolute baldness. Looking at the question "what makes us bald?" from every side, there is one conclusion to be arrived at; it is the hat.

Watch how a man wears his hat, whether daintily on the top of his head—cocked forward impudently and conceitedly—raking aft recklessly—on one side saucily—no matter how, right around where the hat presses his head, there begins the thinness that only requires time to become baldness. But while many admit the hat to be the great hair destroyer, they attribute the result to the unnatural heat which is created between the head and the hat; or to the lack of ventilation there. But the facts, carefully examined, do not warrant any such theory. Those who carefully ventilate their hats with apertures, allowing the air to pass out and in and be constantly renewed, keeping the temperature nearly the same within as without the hat, do not find that the baldness comes later than to those who don't bother about the ventilation or the heat.

The only other cause for baldness that can be associated with the hat is its tightness—the force with which it binds around the head. How many men we see with deep indentures across the forehead, not only flesh deep, but actually showing in the frontal bone, and oftentimes the ridge may be felt with the fingers, in the very bony substance of the skull. Of course this is caused by the tight hat. This hard pressure is not felt because it is so constant, and is applied so early in life that the surface touched by the hat becomes insensible to that pressure. Many may remember, after a long period of illness, or confinement in doors from other causes, how exceedingly uncomfortable a stiff hat is, or any hat, if it be put on tightly. It almost hurts one to wear it, and for the first few days one keeps constantly adjusting it from side to side or front to back, or taking it off upon every opportunity. Even the lightest hat must often be pulled on tightly to prevent its being blown, or shaken, or knocked off, and the spherical shape of the head is such that it acts as a wedge, and a very little pull downwards is followed by a very great pressure. This pressure or binding in a clear and complete circle around the head is what does all the harm. The blood vessels and the other ramifying purveyors of the nutrient principles that supply the scalp are thus either closed, or so compressed that their functions are impaired, and that which should go to the roots of the hair to feed and strengthen it, can not reach beyond the line of pressure, and the hair is literally starved out, dies and falls off, never to grow again because the root is dead or diseased, and all the simples of earth can not restore its life.

Facts will substantiate this theory. Heat and lack of air may and doubtless do assist in destroying the hair, just as a man dying from starvation would go off much quicker in an over-heated or badly-ventilated apartment, but the prime cause is the starvation of the capillary bulbs by cutting off their nutriment.

Women do not get bald as soon as men, and yet they wear hats and bonnets and hoods and wraps of different kind about their heads often of much warmer material and fully as impervious to air. Then they coil their hair in huge masses upon their heads and pin it there, creating great heat, doubtless, and defying the air's penetration. They do more than this. They add to their own hair that of others, and puff it all out, or up, or back with what used to be called "rats," now doubtless known by some other name, "the same with intent to deceive." But they do not bind their heads about with cords, nor do they attach the hat by pressure, but by strings, or elastics, or pins, thus avoiding the compress system. The Indians, until they wear the hat of civilization, do not become bald. It is the hat-wearing nations that lose their hair long before other evidences of decline present themselves, while the turban and fez wearers follow later in life with their shiny pates.

It has been very ingeniously argued that only the excessively clean and fastidious men become bald, and their baldness has been attributed to the frequent changes of the shirt, its often on-putting and off-taking wearing off the hair upon the back of the head by friction. Some weight has been added to this theory during late years by calling attention to the great increase of bald-headed people since the male undergarment of linen was made to open in the back. Those who remember the days of open-fronted shirts will recall how much easier it was to put those garments on and take them off than it is to wiggle into and out of the later fashion of that article of apparel. Moreover the head, in the former case, was poked right through the aperture made for it and there was an end of the process, barring the buttoning. But with the modern style, especially in removing the garment, the back of the head comes in for a good deal of rubbing the wrong way, and it is not a matter of wonder that the idea was conceived that this friction, like the rubbing of harness upon horses, produced a loss of hair. The theory will scarcely work, for we find the dirty man, who changes his clothing only when threatened with arrest by the sanitary officer, becomes bald quite as early as the man who sheds his clothes with every meal.

Probably no effect has been attributed to more causes than baldness. The barbers are blamed for a large share of the baldness of the present day. Well, they do plug up the capillaries with their fats and oils; and they do titillate them with their spirits and their essences; and they do rake them with their harsh brushes and not always smooth-toothed combs; and they seem to lather and shampoo and rub them to death; but those who never visit the barber are about as badly off as the rest—I tell you it is the tight hats that are doing the mischief; and the worst of it all is there is no proposable remedy. We must wear hats— we can't tie them on loosely with a string under our chins—we can't pin them to our hair for there's not hair enough nowadays on ten men's heads to pin one small hat down to. We might wear square hats, made to touch at four spots, and let the circulation have its almost perfect work. In behalf of a bald-headed world, which is yearly growing bald-headeder, I would beg that scientists turn their attention to this great question and restore the hair of coming generations before it come to pass that bald-headity become hereditary, and the human race of the future be hairless as to its cranium.

